# ELSA 2016 Cohort: Alcohol, Tobacco, and Marijuana Use and Their Association with Age of Drug Use Onset, Risk Perception, and Social Norms in Argentinean College Freshmen

**DOI:** 10.3389/fpsyg.2017.01452

**Published:** 2017-08-25

**Authors:** Angelina Pilatti, Jennifer P. Read, Ricardo M. Pautassi

**Affiliations:** ^1^Centro de Investigaciones de la Facultad de Psicológia (CIPSI), Grupo Vinculado al Centro de Investigaciones y Estudios sobre Cultura y Sociedad (CIECS), Consejo Nacional de Investigaciones Científicas y Técnicas (CONICET), Universidad Nacional de Córdoba Córdoba, Argentina; ^2^Department of Psychology, University of Buffalo, Buffalo NY, United States; ^3^Instituto de Investigación Médica M. y M. Ferreyra, Consejo Nacional de Investigaciones Científicas y Técnicas, Universidad Nacional de Córdoba Córdoba, Argentina; ^4^Facultad de Psicología, Universidad Nacional de Córdoba Córdoba, Argentina

**Keywords:** college, substance use, perceived risk, prescriptive norms, age of substance use onset

## Abstract

The transition from high school to college is a high-risk stage for the initiation and escalation of substance use. Substance use and its associated risk factors have been thoroughly described in developed countries, such as the United States, but largely neglected in Argentina, a South American country with patterns of a collectivist culture. The present cross-sectional study describes the occurrence of alcohol, tobacco, and marijuana use and the association between these behaviors and the age of onset of substance use and cognitive (i.e., risk perception) and social (i.e., prescriptive) variables in a large sample of Argentinean college freshmen (*n* = 4083, 40.1% men; mean age = 19.39 ± 2.18 years). The response rate across courses was ≥90% and was similarly distributed across sex. Participants completed a survey that measured substance use (alcohol [with a focus on heavy drinking and binge drinking behaviors], tobacco, and marijuana), age of onset of the use of each substance, perceived risk associated with various substance use behaviors, prescriptive norms associated with substance use, and descriptive norms for alcohol use (AU). The results indicated that AU is nearly normative (90.4 and 80.3% with last year and last month use, respectively) in this population, and heavy drinking is highly prevalent (68.6 and 54.9% with heavy episodic and binge drinking, respectively), especially among those with an early drinking onset (97.8 and 93.6% with last year and last month use and 87.8 and 76.3% with heavy episodic and binge drinking, respectively). The last-year occurrence of tobacco and marijuana use was 36 and 28%, respectively. Early substance use was associated with the greater use of that specific substance. The students overestimated their same-sex friend’s AU, and women overestimated the level of AU of their best male friend. At the multivariate level, all of the predictors, with the exception of the parents’ prescriptive norms, significantly explained the frequency of marijuana and tobacco use and frequency of hazardous drinking. Overall, despite important cultural and contextual differences between Argentina and the United States, our findings suggest that certain vulnerability factors have a similar influence across these cultural contexts.

## Introduction

Several studies indicate a progressive, age-related increase in the consumption of psychoactive substances among Argentinian youth. A nation-wide survey (SEDRONAR, 2014) revealed lifetime alcohol, tobacco, and marijuana use in 51, 5.8, and 21.4% of ≤14 year old Argentinean adolescents, respectively, but these percentages rose to 89, 52.1, and 28.3% among 17–18 year old adolescents. Other Argentinian studies indicated that one-third of college students reported lifetime marijuana use (Pilatti et al., 2014), whereas the last-year occurrence of marijuana use varied between 18% in freshmen (Vera et al., 2015) and 30% in all 5-year college students (Pilatti et al., 2014). Although last-year marijuana use rose to 59% among young adults (Pilatti et al., 2015), the last-month occurrence of tobacco use was fairly similar in college students (33%; Pilatti et al., 2014) and older youth (39.5%; Pilatti et al., 2015). Nearly half of female and male college students reported consuming > 56 and 70 g of pure alcohol, respectively, every time they drank (Pilatti et al., 2014). Between 60% and 71% of college students (Vera et al., 2015; Pilatti et al., 2016a,b) engaged in binge drinking episodes (i.e., the consumption of ≥56 and 70 g of pure alcohol in ≤2 h for women and men, respectively; NIAAA, 2004). Substance use at these ages can interfere with normal brain development (Squeglia et al., 2009; Goriounova and Mansvelder, 2012) and hinder the acquisition of social and educational skills that are needed to achieve independence in adulthood (Masten et al., 2009).

The transition from high school to college is a high-risk stage for the initiation and escalation of substance use (Cho et al., 2015; Derefinko et al., 2016; Skidmore et al., 2016). As explained by different theoretical models, notably the developmental perspective on college AU (Schulenberg and Maggs, 2002), individuals confront new schedules, tasks, and educational and economical responsibilities during this transition and most likely will see their social network profoundly reorganized (Arnett, 2000).

Substance use during this transition has been mostly studied in United States college samples (Ham and Hope, 2003; Grossbard et al., 2010; Quinn and Fromme, 2011; Small et al., 2011; Cho et al., 2015; Derefinko et al., 2016) and not as intensely in other countries, including Argentina. Unknown is whether the risk factors that have been identified in the United States population apply to patterns of substance use in college students who have different cultural backgrounds. The importance of advancing the study of psychological variables in more diverse geographical and cultural groups (Henrich et al., 2010) should not be underestimated. In Argentina, alcohol drinking is a normal part of daily life, and thus this culture can be classified as “wet” (Bloomfield et al., 2003). Argentina also features a recent history of political and economic instability, which has affected alcohol drinking patterns (Munne, 2005). Several cultural differences also exist between the United States and Argentina, and some involve idiosyncratic components of college life. In the United States, the minimum legal age to buy alcohol is 21, whereas the minimum legal age is 18 in Argentina. Thus, unlike their United States counterparts, Argentinian college students spend most of their college years having legal access to alcohol. Also important is that most college students in Argentina attend universities that are close to home, and they live exclusively off-campus. Moreover, United States and Argentinian college students exhibit patterns of individualistic *vs*. collectivist cultures, respectively (Chiou, 2001).

Unknown are the factors that differentiate college students who will engage in regular drug use from those who will not. The perceived risk that is associated with the use of psychoactive substances is one such factor (Johnston et al., 2015). Drugs that are perceived as more dangerous, such as heroin, are less commonly used than those that are perceived as less dangerous, such as marijuana (Maričič, 2013; SEDRONAR, 2014). The perceived risk that is associated with marijuana use distinguished between college students who used marijuana from those who did not (Kilmer et al., 2007; Lopez-Quintero et al., 2011). This evidence, however, is inconclusive. A study of Spanish adolescents found no significant relationship between risk perception and the consumption of various psychoactive substances (Trujillo et al., 2007). Intervening factors may explain these seemingly contradictory patterns. Risk perception is modulated by sex (Petronella-Croisant et al., 2013) and the frequency of drug use (Thornton et al., 2013). Women perceived the use of alcohol, tobacco, and marijuana use as riskier compared with men (Maričič, 2013; Petronella-Croisant et al., 2013), although both sexes had a similar level of risk perception for cocaine and heroin use (Petronella-Croisant et al., 2013). Occasional consumption is perceived as less risky than regular consumption, which in turn is rated as less risky than daily use (Thornton et al., 2013).

The early onset of substance use is another factor that is associated with a heightened risk of developing drug-related problems. Earlier alcohol (Hingson et al., 2006; Dawson et al., 2008), tobacco (Baumeister and Tossmann, 2005; Kendler et al., 2013), and marijuana (Hall and Degenhardt, 2009) consumption is associated with a greater risk of developing substance use disorders. Some authors have postulated that the risk that is associated with substance use onset is substance-specific (i.e., early AU leads to alcohol- but not marijuana-related problems; Ohannessian et al., 2015). Other authors have suggested a broader effect, in which the initiation of use of any substance (e.g., alcohol or tobacco) heightens the risk of using these and other psychoactive substances (Wagner et al., 2005; Hingson et al., 2008; Pilatti et al., 2014).

Social norms (Perkins et al., 1999; Borsari and Carey, 2003) influence drug use directly through the active offering of a substance (Graham et al., 1991; Baer et al., 2001; Wood et al., 2001) and indirectly through descriptive norms (i.e., perceptions about substance use behaviors among relevant social groups) and injunctive norms (i.e., perception of the degree of approval of substance consumption that is held by these social groups; Baer and Carney, 1993; Baer et al., 2001; Neighbors et al., 2011). Young people tend to overestimate the amount and frequency of alcohol consumption of their peers and the perceived approval of binge drinking (Borsari and Carey, 2003).

The association between social norms and substance use has mostly focused on alcohol (Read et al., 2005; LaBrie et al., 2010b; Lewis et al., 2010), although some studies indicated that marijuana (LaBrie et al., 2010a; Buckner, 2013) and tobacco (Zaleski and Aloise-Young, 2013) use approval is significantly associated with their frequency of use. The closeness between the examinee and the reference group significantly modulated these effects (Borsari and Carey, 2003; Lewis et al., 2010).

Very few studies have described the ways in which these factors affect drug use in Argentinean college students, let alone in large samples with adequate sex representation. Men and women use drugs differently (Becker and Hu, 2008; Lev-Ran et al., 2013; Substance Abuse and Mental Health Services Administration [SAMHSA], 2014). Despite recent attempts to foster the visibility of women in epidemiological and basic research (McCullough et al., 2014), most studies continue to equate the role of different risk factors across these populations. Men perceive less risk associated with substance use compared with women (Alvarado et al., 2013; Maričič, 2013; Petronella-Croisant et al., 2013), which may be one explanation for their greater use of substances (Pilatti et al., 2014). Sex-related differences in AU, however, appear to be shrinking (Balodis et al., 2009; Grucza et al., 2009; Keyes et al., 2011), although they still persist for heavy drinking.

The present study included a very large sample (*n* = 4083) of Argentinean college freshmen and separately examined the occurrence of alcohol, tobacco, and marijuana use in women and men and their associations with contextual (i.e., age of onset), cognitive (i.e., risk perception), and social (i.e., prescriptive norms) variables. We also analyzed the relationship between prescriptive norms and perceived risk associated with the consumption of alcohol, tobacco, and marijuana. As mentioned, there is a notorious lack of previous studies analyzing these variables in our target population (i.e., Argentinian freshman). This made our expected outcomes hard to predict. Yet, based on previous work, mostly conducted with United States samples, we outlined a series of preliminary expectations. We expected a large occurrence (i.e., ≥50%) of binge drinking, a behavior that would be expected to be exacerbated among early drinkers (EDs; Hingson et al., 2009). One hypothesis was that early drinking would also affect tobacco and marijuana use (Hingson et al., 2008; Pilatti et al., 2014). We expected greater marijuana use (Suerken et al., 2014) and binge drinking (Johnston et al., 2015; Pilatti et al., 2016b) in men than in women and a negative association between risk perception and substance use (Johnston et al., 2015). With regard to social norms, we expected to find an overestimation of peers’ AU (Borsari and Carey, 2003) and a positive association between perceived approval of substance use and substance involvement (Neighbors et al., 2011).

## Materials and Methods

### Design

This was a cross-sectional study that described the occurrence of substance use in freshman college students and the effect of various risk factors on different indicators of substance use.

### Participants

This study was part of a larger project (Estudio Longitudinal Sobre Alcohol [ELSA]) that assesses alcohol and other drug use in college students in Argentina. Data from the first-wave cohort in 2016 were used in this study. We invited 16 departments of the National University of Cordoba (UNC), Argentina, and 11 accepted. We also invited most sections of National Technological University (UTN) in Córdoba, Argentina. The invitation was sent to and accepted by top officials of each university. The invitation described the study and asked for access to their courses and students for the purpose of administering the survey. UNC is the second largest university in the country, and UTN attracts middle-class high-school graduates from central and northwestern Argentina. These individuals belong to families of large- and medium-sized production farmers, professionals, and local merchants. Thus, they represent a socioeconomic microcosm of the larger Argentinian society. A total of 4122 students fully or partially completed the survey. The response rate across courses was ≥90% and was similarly distributed across sex. Of these surveys, eight cases (five men) were judged as invalid based on extreme inconsistency in the responses, 10 cases (seven men) were almost fully incomplete (i.e., only provided some sociodemographic information), 16 cases (five men) were underage (17 years old), and five cases (three men) were already part of the 2014 ELSA cohort. These 39 cases were thus removed from the analysis. The final sample was composed of 4083 freshmen (40.1% [1639] men), 18–30 years old. The vast majority (96.9%) were between 18 and 25 years of age (mean age, 19.55 ± 2.28 years and 19.28 ± 2.11 years for men and women, respectively). For their participation, the students participated in a raffle in which two cash prizes were given (each ∼USD$72). The sample characteristics are presented in **Table 1**.

**Table 1:** Description of socio-demographic variables as a function of sex.

	Men	Women
**Age**		
Mean Age	19.55 ± 2.28	19.28 ± 2.11
18-25	96.6%	97.1%
26-30	3.4%	2.9%
**University**		
UNC	60.8%	90%
UTN	39.2%	10%
**Employment Status**		
Do not work	81.7%	86.8%
Employed	18.3%	13.2%
**State of origin**		
Cordoba	67%	65.8%
Other	33%	34.2%

*UNC, National University of Cordoba; UTN, National Technological University; State of origin, State where the participants spent most of their lifetime (Cordoba is the city, and the State where the study was conducted).*

### Procedure

The authors administered the survey (paper and pencil format) in the classrooms with the assistance of trained and advanced psychology students. The researchers explained the aim of the study, emphasizing the confidentiality of the data and the voluntary nature of participation. The participants were instructed on how to complete the instruments, and the researchers answered questions concerning survey completion. No personally identifiable information was collected. The students, however, were told that the general aim of the study was to obtain longitudinal data on substance use. Therefore, they were invited to provide their e-mail address and phone number to be contacted in the following stages of the longitudinal study. The students provided written consent before completing the survey. The consent form was on the first page, which could be removed and placed in a separate envelope. Survey administration took ∼35 min, and data collection occurred between April and June 2016. Seven trained and advanced psychology students helped with data entry. These students were part of the research team and were previously trained on ethics associated with data management. Different files were generated to separate the contact information from the survey responses. All of the study procedures were approved by the university’s internal review board, and the protocol was reviewed by the National Agency for Promotion of Science and Technology (FONCyT).

### Measures

#### Dependent Variables

##### Alcohol use

Alcohol use was defined as drinking at least one standard drink (i.e., 14 g pure ethanol; NIAAA, 2004) of any alcoholic beverage. An image described the volume (i.e., in milliliters) of different alcoholic beverages that corresponded to one standard drink. Students reported lifetime, last year, last month, and last week AU and age at first AU (“How old were you the first time you consumed one standard drink or more of any alcoholic beverage?”). Based on previous work (Lee et al., 2012), the students were classified as EDs if they reported first AU by the age of 14 or late drinkers (LDs) if they reported first AU at 15 or older. Two questions asked about the number of standard drinks consumed each day (from Monday to Sunday) in a typical week and each day during the week of heaviest alcohol consumption in the past 3 months.

##### Hazardous alcohol use

We assessed heavy episodic drinking (≥4 and 5 standard drinks in one drinking session for women and men, respectively), binge drinking (≥4 and 5 standard drinks in ≤2 h for women and men, respectively), and drunkenness episodes (Wechsler et al., 2000). The participants indicated the frequency of engaging in heavy episodic and binge drinking episodes within the previous 6 months (from 0 = I do not drink alcohol/I do not drink that amount of alcohol to 8 = four or more times per week). Answers to these two questions were recoded to calculate the number of heavy and binge drinking episodes per month. Three questions asked about the occurrence of drunkenness episodes in their lifetime and in the last 6 months and the number of drunkenness episodes within the previous month.

##### Marijuana use

Based on previous work (Johnston et al., 2015), we asked about lifetime, last year, last month, and last week marijuana use. The participants indicated the age at first marijuana use (“How old were you the first time you used marijuana?”). Based on previous work (Gruber et al., 2012b; Schuster et al., 2016), participants who indicated first marijuana use by the age of 16 were classified as early marijuana users (EMUs), and those who reported first marijuana use at 17 or older were classified as late marijuana users (LMUs). We asked one question to assess the frequency of marijuana use within the previous 6 months (from 0 = I did not use marijuana to 8 ≥ 4 times per week). These answers were recoded to calculate the number of days of marijuana use per month.

##### Tobacco use

We used a similar set of questions to measure lifetime, last year, last month, and last week tobacco use. The participants indicated the age at first tobacco use (at least one whole cigarette). Based on previous work (Morrell et al., 2011), participants who reported first tobacco use by the age of 15 were classified as early smokers (ESs), and those who reported first tobacco use at 16 or older were classified as late smokers (LSs). We asked one question to assess the frequency of tobacco use within the previous 6 months (from 0 = I did not use tobacco to 8 ≥ 4 times per week). These answers were recoded to calculate the number of days of tobacco use per month. The participants also indicated the number of cigarettes they usually consumed per smoking day (0 = I did not use tobacco, 1 = 1–4 cigarettes per day, 2 = 5–9 cigarettes per day, 3 = 10 = 14 cigarettes per day, 4 = 15 = 19 cigarettes per day, and 5 ≥ 20 cigarettes per day).

#### Independent Variables

##### Perceived risk associated with substance use

To assess the perceived risk of using alcohol, tobacco, and marijuana, we adapted questions from the Monitoring the Future study (Johnston et al., 2005) and another study (Yeomans-Maldonado and Patrick, 2015). Specifically, we asked questions about the perceived risk of moderate daily drinking (1–2 standard drinks), heavy episodic drinking (4–5 standard drinks per drinking occasion), drinking 4–5 standard drinks every weekend, drinking enough alcohol to get drunk, combining alcohol and marijuana, and combining alcohol with energy drinks. Three items asked about the perceived risk of daily smoking, smoking on weekends or sometimes per month, and smoking ≥10 cigarettes within a smoking day (e.g., “How much do you think people risk harming themselves [physically, in their health, or in other ways] if they smoke 10 or more cigarettes in one day?”). Four items assessed the perceived risk of using marijuana only once or twice, occasionally (less than once per month), regularly (1–3 times per month), or frequently (once or more per week). Response options ranged from 1 = no risk to 5 = much risk. Answers were summed for each substance, yielding a variable that represented the perception of risk for alcohol (α = 0.75), tobacco (α = 0.76), and marijuana (α = 0.91) use.

##### Injunctive norms

Based on previous work on alcohol (Neighbors et al., 2008b), tobacco (Riou Franca et al., 2009), and marijuana (Neighbors et al., 2008a), we developed three questionnaires to measure the perceived injunctive norms for alcohol, tobacco, and marijuana use (i.e., peer and parental approval/disapproval for the use of each substance).

##### Perceived injunctive norms for alcohol use

Two sets of five questions each measured perceived peer or parental approval of the participants’ AU. The items asked about perceived approval/disapproval of moderate (1–2 standard drinks) and heavy (4-5 standard drinks) daily drinking, drinking 4–5 standard drinks every weekend, drinking enough alcohol to get drunk, and driving a car after drinking alcohol (e.g., “How would your closest friends/parents feel if you drank 4 or 5 standard drinks of alcohol almost daily?”). The response scale ranged from 0 = strong disapproval to 4 = strong approval. The questions concerning parents always had the option to answer “I have no relationship with my parents/I have no parents.” The answers (range, 0–4) to each set of questions were summed, thus yielding two variables in which higher scores reflected a higher level of approval of AU by peers (α = 0.80) and parents (α = 0.76).

##### Perceived injunctive norms for cigarette smoking

Two sets of three questions assessed perceived peer (α = 0.84) and parental (α = 0.89) approval of the participants’ cigarette smoking. The items asked about perceived approval/disapproval of occasional and daily smoking and smoking ≥ 10 cigarettes in a smoking day and used the same response options as those described for AU.

##### Perceived injunctive norms for marijuana use

Two sets of five questions assessed perceived peer (α = 0.94) and parental (α = 0.92) approval/disapproval of lifetime, occasional (less than once per month), regular (1–3 times per month), and frequent (once or more per week) use of marijuana. One question asked about the perceived approval/disapproval of driving a car after using marijuana. The response format was the same as described above.

##### Descriptive norms for alcohol use

Based on the Drinking Norms Rating Form (Baer et al., 1991), we asked participants to estimate the number of standard drinks their closest female friend and closest male friend drank each day in a typical week in the past 3 months. Answers to each of these questions were summed to estimate the perceived weekly drinking by each reference friend. Internal reliabilities were adequate for both best female friend (α = 0.79) and best male friend (α = 0.82) indicators.

### Data Analysis

Descriptive analyses (i.e., frequency, percentage, central tendency, and deviation indices) were conducted for the overall sample and separately for each sex to describe the occurrence of alcohol, tobacco, and marijuana use. Sex differences in tobacco and marijuana use were determined using the *χ^2^* test or Student’s *t*-test for nominal and continuous dependent variables, respectively. We described for the total sample and for each sex the age at which each substance was most likely to be used the first time, the percentage of early and late users, and the percentage of users who began at a specific age (<12 to >20 years). Differences in the occurrence of substance use as a function of age of onset (early, late) for each substance were analyzed using the *χ^2^* test or Student’s *t*-test.

We described the percentage of students who consumed alcohol on each day of the typical or heaviest week of alcohol consumption. Among those who reported alcohol consumption, we calculated the average number of standard drinks consumed on each of these days. These analyses were conducted for the total sample, for early and LDs, and for men and women. Differences in the average number of standard drinks consumed during the typical week and the heaviest week of alcohol consumption as a function of sex and drinking onset (early, late) were analyzed using Student’s *t*-test.

The effect of age of first use on the frequency of use was analyzed separately for each substance using the *χ^2^* test or Student’s *t*-test for nominal and continuous dependent variables, respectively. We also analyzed the effect of age of first use of a given substance (e.g., marijuana) on the occurrence of use of another substance (e.g., tobacco or alcohol). These analyses were conducted in the subsample that had reported lifetime use of that substance (i.e., abstainers or drug-naive participants were excluded from this latter analysis).

A mixed analysis of variance (ANOVA) analyzed own AU, perceived typical same-sex AU, and opposite-sex best friend’s AU. These three indicators of AU were considered within-subject repeated measures, with sex as the between-subjects factor. Tukey’s *post hoc* comparisons were used to analyze significant interactions in the ANOVA.

For each substance, we also evaluated Pearson product-moment correlations between injunctive norms for parents and peers and risk perception associated with the use of that substance and different indicators of substance use. Specifically, for alcohol, the indicators were frequency of heavy and binge drinking, total amount of alcohol consumed during a typical or heaviest week, and total number of drunkenness episodes. For tobacco, the indicators were frequency of tobacco use and number of cigarettes smoked per smoking day. The frequency of marijuana use was the only indicator for that substance.

Multiple regression analyses were performed to evaluate the relationship between a set of independent variables and (a) the frequency of binge drinking, (b) the frequency of tobacco use, and (c) the frequency of marijuana use. Although different indicators of AU could have been chosen, we focused on binge drinking because of its robust association with alcohol-related problems. Separate regressions were run for each of these dependent variables and for each sex. For each analysis, the predictors were early onset of use of the substance under analysis, perceived peer or parental approval of use of the substance, perceived risk of substance use, and (for alcohol only) perceived alcohol consumption of the best female and male friend. Standard multiple regression analyses were used. This method simultaneously added all of the independent variables in the model and yielded regular multiple correlation coefficients (*R^2^*) and standardized regression coefficients.

Descriptive, correlational, and regression analyses were conducted using SPSS 17.0 software. Statistica 7.0 software was used for the ANOVAs. The overall α value was set at 0.05. When appropriate, Bonferroni correction was used to control for multiple comparisons. Specifically, the α for correlations between risk factors and the indicators of substance use was set at 0.00078 (i.e., 0.05/64 comparisons; see **Tables 4, 5**). The α for associations or differences between different indicators of substance use and sex or age of onset was set at 0.003 (i.e., 0.05/19 comparisons; see **Table 2**). It is important to note that the Bonferroni correction is a conservative method to control for type I error (Curtin and Schulz, 1998; Ranstam, 2016), yet can increase type II error. Nonetheless, certain conditions (i.e., large sample size or when the exploration of the data is relatively hypothesis-free) maintain the risk of false negative outcomes (i.e., type II error) at a reasonable level, even after using Bonferroni (Perneger, 1998; Ranstam, 2016). In these analyses in which Bonferroni was applied, effect sizes were estimated to provide further information on the magnitude of the effects found. Effects sized were interpreted as described by Cohen (1988, 1992a,b).

**Table 2:** Occurrence of alcohol, tobacco and marijuana use: for the total sample and as a function of sex and age at each substance onset.

	Total sample					Ever alcohol users				Ever tobacco users				Ever marijuana users			
	Total	Men	Women	χ^2^/t	EfS	LD	ED	χ^2^/t	EfS	LS	ES	χ^2^/t	EfS	LUM	EUM	χ^2^/t	EfS
**Alcohol**																	
Lifetime	94.6	96.2	93.6	13.50^∗^	0.06	100	100	–	–	99.5	99.3	0.49	0.01	99.6	99.5	0.18	0.01
12M	90.4	92.4	89	13.41^∗^	0.06	94.5	97.8	19.32^∗^	0.07	96.5	96.3	0.10	0.01	96.9	97	0.01	0.00
30D	80.3	84.9	77.1	37.53^∗^	0.10	81.2	93.6	95.82^∗^	0.15	89.9	91.3	1.36	0.02	91.9	93	0.63	0.01
7D	50.4	59.6	44.2	92.95^∗^	0.15	46.2	70.3	185.68^∗^	0.21	61.5	67.4	7.41	0.04	69	73.4	3.32	0.03
HED	68.6	76	63.7	68.33^∗^	0.13	66.7	87.8	179.41^∗^	0.21	79.8	83.6	4.75	0.03	83.1	87.5	5.23	0.04
Binge	54.9	64.1	48.8	91.68^∗^	0.15	50.8	76.3	211.74^∗^	0.23	65.8	70.6	5.27	0.04	68.6	77	12.28^∗^	0.05
**Drunkenness**																	
Lifetime	70.1	75.7	66.4	40.57^∗^	0.10	67.9	89.6	198.04^∗^	0.22	83.8	87.7	6.01	0.04	90.2	93.5	4.95	0.03
6M	44.4	51.1	39.9	49.38^∗^	0.11	39.8	64.3	188.99^∗^	0.22	53.3	57.5	3.46	0.03	58.1	68.4	15.43^∗^	0.06
30D	23.9	29.3	20.3	43.57^∗^	0.10	20.1	37.7	130.23^∗^	0.18	29	36.5	12.68^∗^	0.06	32.5	44.8	22.55^∗^	0.07
Q D 30D	0.50 ± 1.32	0.69 ± 1.63	0.38 ± 1.04	7.47^∗^	0.12	0.38 ± 1.08	0.89 ± 1.79	10.79^∗^	0.17	0.57 ± 1.24	0.90 ± 1.86	4.85^∗^	0.08	0.71 ± 1.59	1.08 ± 1.85	3.97^∗^	0.06
**Tobacco**																	
Lifetime	51.3	52.7	50.4	2.07	0.02	47.5	69.5	154.76^∗^	0.19	100	100	–		80	87	12.13^∗^	0.05
12M	36.3	36	36.5	0.09	0.00	32.1	52.9	146.06^∗^	0.19	70	72.1	1.10	0.02	60.6	67.7	7.75	0.04
30D	27.4	27.4	27.4	0.00	0.00	23.6	41.1	122.75^∗^	0.17	48.3	61.4	34.51^∗^	0.10	48.8	57.3	10.17^∗^	0.05
7D	21.4	21.2	21.6	0.00	0.00	18.4	32.6	92.10^∗^	0.15	34.7	52.5	64.66^∗^	0.13	38.4	48.2	13.87^∗^	0.06
Q C Day	1.75 ± 3.64	1.91 ± 3.98	1.64 ± 3.40	2.24	0.04	1.44 ± 3.28	2.81 ± 4.23	10.47^∗^	0.17	2.68 ± 3.56	4.56 ± 5.38	9.50^∗^	0.15	3.08 ± 4.44	4.14 ± 5.23	4.13^∗^	0.06
**Marijuana**																	
Lifetime	36	45.1	30	96.42^∗^	0.15	30.6	55.8	213.53^∗^	0.23	49.9	70.5	86.99^∗^	0.15	100	100	–	
12M	27.5	35.5	22.1	88.59^∗^	0.15	22.1	45.4	207.55^∗^	0.23	37.9	55	58.47^∗^	0.12	74.2	79.7	5.89	0.04
30D	17.5	23.4	13.6	64.14^∗^	0.13	13	31.4	176.44^∗^	0.21	22.7	39.9	70.06^∗^	0.13	40.2	61.3	62.49^∗^	0.12
7D	9.8	13.6	7.3	44.28^∗^	0.10	7.1	18	101.27^∗^	0.16	12.1	23.9	49.54^∗^	0.11	18.4	40.7	87.09^∗^	0.15

*LD, late drinkers; ED, early drinkers; LS, late smokers; ES, early smokers; LUM, late users of marijuana; EUM, early users of marijuana; Lifetime, lifetime use; 12M, last year occurrence; 30D, last month occurrence; 7D, last week occurrence; HED, heavy episodic drinking (4/5 standard drinks in one drinking session); Binge, binge drinking (4/5 standard drinks in ≤2 h); Q D 30D, number of drunkenness episodes within the previous month; Q C Day, quantity of cigarettes consumed per smoking day; EfS, effect size. ^∗^*p* ≤ 0.003.*

We consider other alternatives to analyze the data without inflating the risk of a type I error. We could, instead of the multiple bivariate associations, have used a principal component analysis. This approach, however, would have seriously diminished obtaining a detailed analysis of these relationships (Curtin and Schulz, 1998).

Descriptive values and statistical notations (i.e., *χ^2^* values, *F*-values, *p*-values for each analysis, etc.) for most of the inferential analyses are shown in the tables.

## Results

### Descriptive Results

#### Alcohol Use

Alcohol was by far the most consumed substance. The vast majority of the participants reported consuming at least one standard unit of alcohol in their lifetime and within the previous year and previous month. Almost 70 and 55% of the sample engaged in at least one episode of heavy episodic drinking and binge drinking, respectively, within the previous 6 months. Despite the elevated occurrence of heavy and binge drinking, less than half of the students reported drunkenness episodes within the same timeframe. These results are presented in **Table 2**. As shown in **Table 3**, ∼33 and 18.3% of the sample engaged in heavy episodic drinking and binge drinking, respectively, at least once per week.

**Table 3:** Frequency of binge and heavy drinking and frequency of tobacco and marihuana use: for the total sample and as a function of sex.

	Alcohol											
	HED			Binge			Tobacco			Marijuana		
	Total	Men	Women	Total	Men	Women	Total	Men	Women	Total	Men	Women
No use	31.5	24.1	36.4	45.1	35.9	51.2	67.8	67.5	68	76.2	69	81.1
≤1 M	10.5	10.7	10.3	13.4	15.8	11.8	5.3	5.2	5.3	7.9	10	6.6
1 M	8.8	8.2	9.2	11.5	11.9	11.3	2.1	2.8	1.7	2.6	3.5	2.1
2 M	9.7	9.8	9.7	7	8	6.3	2.5	2.7	2.4	3	3.4	2.7
3 M	7	6.6	7.3	4.7	4.7	4.7	2.1	1.9	2.3	2.1	2.3	2
1 W	17.2	19.5	15.7	11.3	14.6	9.1	3.4	4.1	2.9	2.2	2.8	1.8
2 W	11.9	15.9	9.2	5.7	7.3	4.7	2.7	2.3	3	1.9	2.8	1.4
3 W	2.6	4.1	1.6	1.1	1.5	0.7	3	3	2.9	1.5	2.3	1
≥4 W	0.7	1	0.5	0.2	0.3	0.2	11.1	10.4	11.5	2.4	3.9	1.4

*HED, heavy episodic drinking, defined as the consumption of ≥4/5 standard drinks in one drinking session (women/men); Binge drinking = defined as the consumption of ≥4/5 standard drinks in ≤2 h (women/men).*

#### Tobacco Use

Half of the sample indicated lifetime use of tobacco, with approximately one-third of the students reporting smoking cigarettes within the previous year (**Table 2**). Most current tobacco users exhibited daily smoking (**Table 3**).

#### Marijuana Use

Marijuana use had the lowest prevalence (**Table 2**) compared with alcohol and tobacco. The majority of marijuana users reported low-frequency patterns of consumption (**Table 3**). The percentage of students who reported a high frequency of marijuana use (≥3 times per week; 3.9%) was greater than the percentage of students who exhibited a similar frequency of binge drinking (1.3%).

#### Alcohol Use during the Typical and Heaviest Weeks of Alcohol Consumption

The mean number of standard drinks of alcohol that were consumed during the typical and heaviest weeks were 7.39 ± 9.12 and 13.75 ± 17.06, respectively. The lowest percentages of students reported drinking Sunday to Wednesday (range, 2.3–9.6%), whereas the highest percentages of students reported drinking on Friday and Saturday (50.8 and 77.1%, respectively). A similar pattern was found during the heaviest week of consumption, although the percentages were more spread out across days, with an increasing number of students who reported drinking on weekdays (e.g., drinking on Wednesday and Thursday increased from 5.9 and 12.8% to 15.7 and 28.4% in the typical and heaviest weeks, respectively). The number of standard drinks consumed each day of the typical and heaviest weeks also changed during the week. Specifically, in a typical week, the participants reported drinking an average of around two standard drinks each day from Sunday to Wednesday, three drinks on Thursday, and around five drinks on Friday and Saturday. In the heaviest week of alcohol consumption, they reported drinking an average of about four standard drinks each day from Sunday to Wednesday, five drinks on Thursday, and between 6.55 and 7.55 drinks on Friday and Saturday, respectively.

### Onset of Substance Use

#### Age of Onset of Alcohol Drinking

Among lifetime drinkers, the majority (70.1%) reported the first consumption of at least one standard drink between 14 and 16 years of age, with nearly 60% of all drinkers doing so by the age of 15. Only 2% of the drinkers reported first AU at 18 or older. Nearly 30% of the drinkers (29.9%) were classified as EDs, and the rest were classified as LDs. Among lifetime drinkers, the mean age of onset of AU was 15.21 ± 1.58 years. As expected, EDs reported a significantly lower age of drinking onset (mean = 13.44 ± 0.98 years) than LDs (15.97 ± 1.11 years; *t* = 66.57, *p* ≤ 0.001).

#### Age of Onset of Tobacco Use

Among lifetime smokers, the age at first tobacco use was concentrated within the age range of 15–17 years (60.2%). Forty percent of the smokers reported first tobacco use by the age of 15, and 16.3% of them began at 18 or older. Among lifetime smokers, the mean age of first tobacco use was 15.83 ± 1.95 years, and ESs (13.96 ± 1.26 years) reporting first tobacco use at a significantly younger age than LSs (17.08 ± 1.18 years; *t* = 57.60, *p* ≤ 0.001).

#### Age of Onset of Marijuana Use

Among lifetime users of marijuana, the majority reported first marijuana use within the age range of 16–18 years (65.9%). Nearly 19% reported first use by the age of 15, and 34.1% of them began at 18 or older. Among lifetime marijuana users, the mean age of first marijuana use was 17.03 ± 2.02 years. As expected, EMUs reported a significantly younger age of first marijuana use (15.33 ± 0.91 years) than LMUs (18.23 ± 1.70 years; *t* = 38.18, *p* ≤ 0.001).

### Group Differences

#### Sex Differences

Men reported a significantly higher occurrence and average number of general and hazardous (heavy, binge, and drunkenness episodes) AU compared with women. Men had a significantly higher occurrence of marijuana use compared with women. No sex differences were found for tobacco. These results are presented in **Tables 2, 3**. Men also reported drinking a significantly greater amount of alcohol (standard units) during the typical (10.21 ± 11.21) and heaviest (19.75 ± 21.25) weeks of AU compared with women (mean_typical_ = 5.49 ± 6.75; mean_heaviest_ = 9.72 ± 11.94).

Men and women had a similar pattern of ages of alcohol, tobacco, and marijuana initiation. Across sex, most of the participants exhibited (a) first AU by the age of 14–16, (b) first tobacco use by the age of 15–17, and (c) first marijuana use by the age of 16–18. Despite this general trend, men reported a slightly but significant lower mean age of alcohol onset (mean_men_ = 14.95 ± 1.68 years; mean_women_ = 15.39 ± 1.47 years; *t* = 8.62, *p* ≤ 0.001), tobacco onset (mean_men_ = 15.65 ± 2.01 years; mean_women_ = 15.96 ± 1.90 years; *t* = 3.46, *p* ≤ 0.001), and marijuana onset (mean_men_ = 16.77 ± 1.98 years; mean_women_ = 17.28 ± 2.03 years; *t* = 4.80, *p* ≤ 0.001) compared with women. The percentages of men who were classified as early users of alcohol (36.5%) and marijuana (46.4%) but not tobacco (42.5%) were significantly higher than the percentages of women (alcohol = 25.3%; tobacco = 38.4%; marijuana = 36.4%; *χ^2^*_alcohol_ = 54.23, *p* ≤ 0.001; *χ^2^*_tobacco_ = 3.50, *p* = 0.061; *χ^2^*_marijuana_ = 15.08, *p* ≤ 0.001).

#### Age at Drinking Onset and Use of Alcohol, Tobacco, and Marijuana

Early drinkers reported significantly greater AU for all the drinking indicators than their peers who began drinking alcohol at older ages. All of the tobacco and marijuana use indicators were significantly greater in EDs than in LDs. These results are presented in **Table 2**. EDs also reported drinking a significantly greater amount (standard units) of alcohol during the typical (11.73 ± 11.51) and heaviest (22.69 ± 22.10) weeks of AU compared with LDs (mean_typical_ = 6.16 ± 7.39; mean_heaviest_ = 11.12 ± 13.18).

#### Age at Tobacco Onset and Use of Tobacco, Alcohol, and Marijuana

Early smokers reported a significantly higher occurrence of last month and last week but not last year tobacco use compared with LSs. ESs also smoked a greater number of cigarettes per smoking day compared with LSs. ESs reported a significantly higher occurrence of last month drunkenness compared with LSs. ESs reported a significantly greater occurrence of all of the indicators of marijuana use compared with LSs. These results are presented in **Table 2**.

#### Age at Marijuana Onset and Use of Marijuana, Alcohol, and Tobacco

Early marijuana users reported a significantly higher occurrence of all indicators of marijuana use compared with LMUs. EMUs reported a significantly greater use of tobacco and a greater occurrence of binge drinking and drunkenness episodes compared with LMUs. These results are presented in **Table 2**.

Effects sizes (**Table 2**) for the associations between substance use and sex were low for marihuana and alcohol (i.e., between 0.10 and 0.15 and between 0.06 and 0.15, respectively), whereas those for tobacco ranged between 0.0 and 0.04. The effect size of early drinking onset on subsequent alcohol (0.07–0.23), tobacco (0.15–0.19) or marihuana (0.16–0.23) use was larger than the effect of early tobacco or early marihuana use on subsequent use of each of these substances. The effect of early tobacco onset was larger for subsequent marihuana use (0.11–0.15) than for subsequent use of tobacco (0.02–0.15) or for AU (0.01–0.08). The effect of early use of marihuana was larger for the subsequent use of marihuana (0.04–0.15) than for the subsequent use of tobacco (0.04–0.06) or use of alcohol (0.0–0.07).

#### Own vs. Perceived Amount of Alcohol Use as a Function of Sex

**FIGURE 1:**
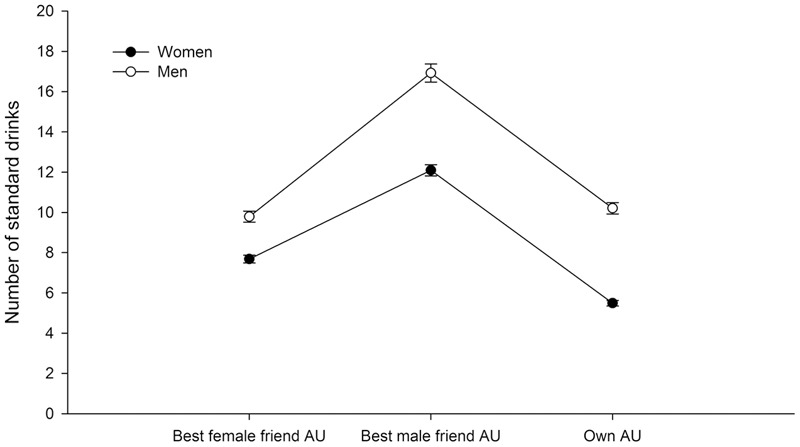
Alcohol use (AU), expressed as the mean number of standards drinks consumed in a typical week, by each participant (own AU) and perceived number of standards drinks consumed in a typical week by the best female friend and best male friend. The vertical lines indicate the standard error of the mean. Refer to the text for significant differences that were found in the statistical analysis.

The mixed ANOVA revealed a significant Sex × Indicator of Alcohol Use interaction (*F*_2,7342_ = 39.40, *p* ≤ 0.001; **Figure 1**). The *post hoc* analyses indicated significant sex differences in the total amount of own AU, in which men drank more alcohol than women within a typical week of alcohol consumption. Moreover, women perceived that either their best female friend or best male friend drank significantly more heavily than they did. Men perceived that they drank a significantly lower amount of alcohol than their best male friend but as much as their best female friend.

### Correlations

#### Perceived Risk Associated with Substance Use

##### Alcohol use

The perceived risk of AU was significantly and negatively correlated with all indicators of AU. Women (Pearson *r_s_* = -0.19 to -0.32) and men (Pearson *r_s_* = -0.23 to -0.34) presented a similar pattern of correlations (**Table 4**).

**Table 4:** Correlations between perceived risk, social and injunctive norms (parents and friends) with alcohol use.

	TW FF	TW MF	FD	MD	IN F	IN P	PRA	F HED	F B	TW	IW	Q D E
TW FF		0.77^∗†^	0.33^∗‡^	0.24^∗⁞^	0.23^∗⁞^	0.12^∗⁞^	-0.19^∗⁞^	0.48^∗‡^	0.44^∗‡^	0.59^∗†^	0.63^∗†^	0.20^∗⁞^
TW MF	0.78^∗†^		0.25^∗⁞^	0.28^∗⁞^	0.31^∗‡^	0.17^∗⁞^	-0.23^∗⁞^	0.56^∗†^	0.49^∗‡^	0.71^∗†^	0.72^∗†^	0.26^∗⁞^
FD	0.30^∗‡^	0.27^∗⁞^		0.56^∗†^	0.30^∗‡^	0.14^∗⁞^	-0.24^∗⁞^	0.26^∗⁞^	0.24^∗⁞^	0.23^∗⁞^	0.24^∗⁞^	0.15^∗⁞^
MD	0.22^∗⁞^	0.28^∗⁞^	0.69^∗†^		0.31^∗‡^	0.11^∗⁞^	-0.23^∗⁞^	0.26^∗⁞^	0.22^∗⁞^	0.25^∗⁞^	0.27^∗⁞^	0.14^∗⁞^
IN F	0.32^∗‡^	0.30^∗‡^	0.37^∗^	0.31^∗‡^		0.37^∗‡^	-0.40^∗‡^	0.35^∗‡^	0.34^∗‡^	0.30^∗‡^	0.30^∗‡^	0.23^∗⁞^
IN P	0.10^∗⁞^	0.10^∗⁞^	0.09^∗⁞^	0.02	0.28^∗⁞^		-0.33^∗‡^	0.18^∗⁞^	0.21^∗⁞^	0.18^∗⁞^	0.17^∗⁞^	0.14^∗⁞^
PRA	-0.21^∗⁞^	-0.19^∗⁞^	-0.22^∗⁞^	-0.17^∗⁞^	-0.38^∗‡^	-0.27^∗‡^		-0.34^∗‡^	-0.31^∗^	-0.30^∗‡^	-0.30^∗‡^	-0.23^∗⁞^
F HED	0.51^∗†^	0.49^∗‡^	0.32^∗‡^	0.24^∗⁞^	0.35^∗‡^	0.17^∗⁞^	-0.32^∗‡^		0.77^∗†^	0.77^∗†^	0.70^∗†^	0.38^∗‡^
F B	0.43^∗‡^	0.41^∗‡^	0.27^∗⁞^	0.18^∗⁞^	0.31^∗‡^	0.15^∗⁞^	-0.28^∗⁞^	0.78^∗†^		0.65^∗^	0.62^∗†^	0.37^∗‡^
TW	0.64^∗†^	0.62^∗†^	0.29^∗⁞^	0.22^∗⁞^	0.33^∗‡^	0.16^∗⁞^	-0.28^∗⁞^	0.75^∗†^	0.63^∗†^		0.84^∗†^	0.33^∗‡^
IW	0.67^∗†^	0.66^∗†^	0.29^∗⁞^	0.22^∗⁞^	0.33^∗‡^	0.16^∗⁞^	-0.30^∗‡^	0.72^∗†^	0.62^∗†^	0.85^∗†^		0.31^∗‡^
Q D E	0.27^∗⁞^	0.25^∗⁞^	0.16^∗⁞^	0.11^∗⁞^	0.21^∗⁞^	0.09^∗⁞^	-0.19^∗⁞^	0.40^∗‡^	0.37^∗‡^	0.36^∗‡^	0.36^∗‡^	

*The upper triangle presents results among men. The lower triangle presents results among women; TW FF, Perceived weekly drinking for the closest female friend; TW MF, Perceived weekly drinking for the closest male friend; FD, perceived quantity of female drinkers; MD, perceived quantity of male drinkers; IN F = perceived peers’ norms for alcohol use; perceived risk for alcohol use; IN P, perceived parents’ norms for alcohol use; PRA, perceived risk for alcohol use; F HED, frequency of heavy drinking; F B, frequency of binge drinking; TW, total alcohol consumption during a typical week; IW, total alcohol consumption during the heaviest week of alcohol consumption; QED, total number of drunkenness episodes. *^∗^p ≤* 0.001; The ^⁞,‡, †^ signs indicate low, medium and large effect sizes.*

##### Tobacco use

The perceived risk of tobacco use was significantly and negatively correlated with the frequency and amount of tobacco use, a pattern that was fairly similar across men (*r_s_* = -0.19 to -0.16) and women (Pearson *r_s_* = -0.22 to -0.19; **Table 5**).

**Table 5:** Correlations between perceived risk and injunctive norms (parents and friends) with tobacco and marijuana use.

	Tobacco					Marijuana				
	PRT	IN P	IN F	FT	QC		PRM	IN P	IN F	FM
PRT		-0.13^∗⁞^	-0.22^∗⁞^	-0.19^∗⁞^	-0.16^∗⁞^	PRM		-0.27^∗⁞^	-0.51^∗†^	-0.42^∗‡^
IN P	-0.17^∗⁞^		0.38^∗‡^	0.25^∗⁞^	0.21^∗⁞^	IN P	-0.25^∗⁞^		0.36^∗‡^	0.35^∗‡^
IN F	-0.25^∗⁞^	0.33^∗‡^		0.29^∗⁞^	0.29^∗⁞^	IN F	-0.55^∗†^	0.29^∗⁞^		0.49^∗‡^
FT	-0.22^∗⁞^	0.30^∗‡^	0.37^∗‡^		0.75^∗†^	FM	-0.35^∗‡^	0.25^∗⁞^	0.43^∗‡^	
QC	-0.19^∗⁞^	0.29^∗⁞^	0.32^∗‡^	0.74^∗†^						

*PRT, perceived risk for tobacco use; IN P, perceived parents′ norms for tobacco use; IN F, perceived peers′ norms for tobacco use; FT, frequency of tobacco use; QC, quantity of cigarettes consumed per smoking day; PRM, perceived risk for marijuana use; IN P, perceived parents′ norms for marijuana use; IN F, perceived peers′ norms for marijuana use; FM, frequency of marijuana use. The upper triangle presents results among men. The lower triangle presents results among women. ^∗^p ≤ 0.001; The ^⁞,‡,†^ signs indicate low, medium and large effect sizes.*

##### Marijuana use

The perceived risk of marijuana use was negatively and significantly correlated with the frequency of marijuana use. The size of this correlation was the highest among the three substances and greater among men (Pearson *r* = -0.42) than among women (Pearson *r* = -0.35; **Table 5**).

#### Injunctive Norms

##### Alcohol use

The perceived levels of both peer and parental approval of AU were positively and significantly correlated with hazardous alcohol drinking (heavy and binge drinking and number of drunkenness episodes) and the quantity of alcohol consumed during the typical and heaviest weeks of alcohol intake. The effect size was stronger for perceived peer approval (Pearson *r_s_* = 0.21 to 0.35) than for perceived parental approval (Pearson *r_s_* = 0.09 to 0.21). Although men and women presented very similar patterns of correlations, the association between drunkenness episodes and perceived parental approval was quite low among women (Pearson *r* = 0.09; **Table 4**).

##### Tobacco use

Students who perceived greater peer and parental approval of tobacco use reported a significantly higher frequency and amount of tobacco use, although the effect was greater for perceived peer approval. In contrast to alcohol, the association between parents’ norms and tobacco use were stronger for women (Pearson *r_s_* = 0.30 and 0.29 for frequency and amount, respectively) than for men (Pearson *r_s_* = 0.25 and 0.21 for frequency and amount, respectively; **Table 5**).

##### Marijuana use

The perceived levels of peer and parental approval of marijuana use were positively and significantly correlated with the frequency of marijuana use. The effect size for peers (Pearson *r* = 0.49 and 0.43 for men and women, respectively) was stronger than for parents (Pearson *r* = 0.35 and 0.25 for men and women, respectively; **Table 5**).

#### Descriptive Norms for Alcohol Use

All indicators of own AU were positively and significantly correlated with descriptive norms (**Table 4**). Among women, the size of the associations was similar, regardless of the sex of the best friend. Among men, the associations that involved a male friend were stronger than those that involved a female friend.

#### Injunctive Norms and Perceived Risk Associated with Substance Use

The level of approval of alcohol (**Table 4**), tobacco (**Table 5**), and marijuana (**Table 5**) use was negatively and significantly associated with the perceived risk of using each of these substances. Across sex, the size of the correlations was stronger for peers than for parents as the reference group.

The effect sizes between the independent variables and AU were larger for those variables that involved the peers. More in detail, many of the effect sizes of the correlations between descriptive norms and AU were large (i.e., between 0.20 and 0.72). The effect sizes of the correlations between injunctive norms and AU were larger when the reference group was the peers (i.e., between 0.21 and 0.35) than when the reference group was the parents (i.e., between 0.09 and 0.21). The effect sizes of the correlations between peers’ injunctive norms and tobacco or marihuana use were medium and close to large, respectively; whereas those involving the parents were medium for both substances. The effect size of the correlation between perceived risk and substance use was close to large for marihuana, medium for alcohol (i.e., most around 0.30) and low for tobacco (see **Tables 4, 5**).

### Regression Analyses

#### Frequency of Binge Drinking

Among women, the independent variables accounted for 27% of the variance of binge drinking (***F*_change6_**_,1954_ = 120.44, *p* ≤ 0.001). All of the predictors, with the exception of the perceived parental approval of AU, significantly explained the frequency of binge drinking. Early drinking onset (β = -0.14, *t* = -7.07, *p* ≤ 0.001), perceived peer approval of AU (β = 0.11, *t* = 4.93, *p* ≤ 0.001), perceived amount of alcohol consumption of the best female friend (β = 0.20, *t* = 6.53, *p* ≤ 0.001) or male friend (β = 0.17, *t* = 5.61, *p* ≤ 0.001), and perceived risk (β = -0.13, *t* = -6.26, *p* ≤ 0.001) were significantly associated with a higher frequency of binge drinking. Similar results were found among the subsample of men. Five of the six predictors significantly explained 33% of the variance (*F*_change6,1310_ = 109.81, *p* ≤ 0.001). Early drinking onset (β = -0.11, *t* = -4.57, *p* ≤ 0.001), perceived peer approval of AU (β = 0.13, *t* = 4.84, *p* ≤ 0.001), perceived amount of alcohol consumed by the best female friend (β = 0.17, *t* = 4.55, *p* ≤ 0.001) or male friend (β = 0.26, *t* = 6.95, *p* ≤ 0.001), and perceived risk (β = -0.14, *t* = -5.40, *p* ≤ 0.001) but not perceived parental approval of AU were significantly associated with a higher frequency of binge drinking.

#### Frequency of Tobacco Use

Among women, a significant model emerged for the four independent variables, with an *R^2^* that accounted for 21% of the variance in the self-reported frequency of tobacco use (*F*_change4,1165_ = 74.35, *p* ≤ 0.001). Early tobacco use (β = -0.18, *t* = -6.86, *p* ≤ 0.001), perceived peer approval of tobacco use (β = 0.27, *t* = 9.78, *p* ≤ 0.001), perceived parental approval of tobacco use (β = 0.15, *t* = 5.44, *p* ≤ 0.001), and perceived risk (β = -0.12, *t* = -4.34, *p* ≤ 0.001) were significantly associated with more frequent tobacco use. Among men, the total explained variance (*R^2^* = 0.14) was somewhat lower compared with women (*F*_change4,781_ = 31.82, *p* ≤ 0.001). Early tobacco onset (β = -0.11, *t* = -3.20, *p* ≤ 0.05), perceived peer approval of tobacco use (β = 0.18, *t* = 4.85, *p* ≤ 0.001), and perceived parental approval of tobacco use (β = 0.16, *t* = 4.29, *p* ≤ 0.001) were significantly and positively associated with a higher frequency of smoking cigarettes. Perceived risk was negatively associated with tobacco use (β = -0.13, *t* = -3.74, *p* ≤ 0.001).

#### Frequency of Marijuana Use

Among women, the independent variables explained 25% of the total variance of the frequency of marijuana use (*F*_change4,687_ = 58.06, *p* ≤ 0.001). Early marijuana onset (β = -0.11, *t* = -3.18, *p* ≤ 0.01), perceived peer approval of marijuana use (β = 0.31, *t* = 8.04, *p* ≤ 0.001), perceived parental approval of marijuana use (β = 0.10, *t* = 2.77, *p* ≤ 0.01), and perceived risk (β = -0.19, *t* = -4.95, *p* ≤ 0.001) were significantly associated with the frequency of marijuana use. Among men, the variables explained 36% of the total variance (*F*_change4,659_ = 92.60, *p* ≤ 0.001). Early marijuana use (β = -0.18, *t* = -5.51, *p* ≤ 0.001), perceived peer approval of marijuana use (β = 0.32, *t* = 8.45, *p* ≤ 0.001), perceived parental approval of marijuana use (β = 0.15, *t* = 4.50, *p* ≤ 0.001), and perceived risk associated with marijuana use (β = -0.22, *t* = -6.14, *p* ≤ 0.001) were significantly associated with more frequent marijuana use.

## Discussion

The present study described alcohol (with a focus on binge and heavy episodic drinking), marijuana, and tobacco use in a large sample (*n* = 4083) of Argentinean college freshmen. We also assessed (a) the modulation of these patterns by personal beliefs about the risk of use of these substances (in varying degrees of intensity), (b) the modulation of these patterns by the perception of their use and approval by peers and parents, and (c) whether the onset of use of a given substance influences the use of that substance or the other substances. Recent studies (Keyes et al., 2011; Slade et al., 2016) suggest that the gap in drug use between men and women is shrinking. An important aim of the present study was to analyze sex differences in these effects. Previous studies by our group included smaller, albeit substantially similar, samples and found that the age of onset of AU was an important facilitator of hazardous drinking behaviors. Therefore, we assessed the generality of this effect of age of onset for other substances.

As expected, lifetime and last year use of alcohol was normative (i.e., 94.6 and 90.4%, respectively), and only 2% of ever-drinkers drank a full drink at or after the legal age (i.e., ≥18 years, in Argentina). These percentages are greater than those that were reported for United States college students in the Monitoring the Future study (Johnston et al., 2015). One caveat of this comparison is that the Monitoring the Future study defined college students as respondents who were 1–4 years beyond high school, whereas all of the respondents in the present study were college freshmen.

An important finding was the higher occurrence of hazardous AU. Nearly 70 and 55% of the students reported heavy episodic or binge drinking in the last 6 months. Moreover, approximately 33 and 20% of the sample engaged in heavy episodic drinking and binge drinking, respectively, on a weekly basis. These figures are somewhat similar to those reported by the Monitoring the Future study, although they asked about heavy drinking within the previous 2 weeks. Tobacco use (51.3 and 36.3% lifetime and last year use, respectively) and marijuana use (36.0 and 27.5% lifetime and last year use, respectively) was lower than AU. The figures for marijuana use were markedly lower than those reported by the Monitoring the Future study in the United States (50.4 and 38.0% lifetime and last year use, respectively), although this comparison should be framed within the context of the aforementioned difference in the years of college enrollment. Unlike alcohol and marijuana users, most of the tobacco users in the present study reported almost daily tobacco consumption. This underscores the addictive liability of nicotine. A previous study found that 21% of those who had ever tried nicotine became dependent on the substance compared with 11 and 4% of those who had ever used alcohol or marijuana, respectively (Schramm-Sapyta et al., 2009).

Last year tobacco use was similar to recent studies that were conducted with college freshmen in Argentina (Vera et al., 2015) and the United States (Suerken et al., 2014). An interesting comparison can be made concerning another nationwide Argentinean study (SEDRONAR, 2010). Last year tobacco use in the present study was similar to (although somewhat lower than [5.5%]) SEDRONAR, but last year marijuana use in our sample (35.5 and 22.1% for men and women, respectively) almost doubled compared with reports by SEDRONAR 6 years ago. A recent study of United States college students (Suerken et al., 2014) reported a 29.8% prevalence of marijuana use in the last 6 months. The Monitoring the Future study of senior high-school students in the United States reported a gradual increase in the last-year use of marijuana from 2006 to 2011, but this increase leveled off afterward (Johnston et al., 2015). Altogether, these results suggest a steady increase in recent (i.e., last year and last 6 months) use of marijuana among late adolescents, although regional differences are likely to occur, particularly when focusing on specific patterns of marijuana use. The prevalence of intensive marijuana use (i.e., in the last 7 days) in the present study was 13.6 and 7.3% for men and women, respectively, which is ostensibly lower compared with college students in Spain (22.2 and 20.0%, respectively; Moure-Rodríguez et al., 2016). The apparent increase in marijuana use among Argentinean adolescents is concerning. Marijuana use has been associated with lower academic performance, a higher risk of dropping out of college (Suerken et al., 2014), and the use of other illegal drugs (Babor et al., 2010).

The analysis of sex differences in the frequency of binge and heavy drinking and frequency of tobacco and marijuana use revealed an interesting pattern. Men and women exhibited a fairly similar prevalence of these behaviors when focusing on less-than-weekly use (i.e., once, twice, or three times per month). After this threshold of use, the frequency of alcohol and marijuana but not tobacco use was an average of two-times higher in men than in women (e.g., the biweekly use of marijuana was 1.4 and 2.8% for men and women, respectively). This reflects the closing gap between sexes in drug use (Wallace et al., 2003; Keyes et al., 2011; Slade et al., 2016), which may be more conspicuous among those who do not present patterns of heavy drug use (Corbin et al., 2008). A previous study (Johnston et al., 2006) found that the narrowing gap between sexes in AU was not the same for all ethnic groups. Latino youths exhibited the largest sex gap in the 30-day prevalence of AU (11%) compared with American Caucasians, Asian Americans, and American Indians. Sex differences within Latino samples were also notable, with a peak of 14% among those of Mexican ancestry, followed by 10% among Puerto Rican Americans. Individuals from other Latin American countries presented a 9% sex gap (Johnston et al., 2006), which is similar to the 7.8% sex gap that was found in our sample of Argentinian college students.

An interesting comparison of alcohol consumption patterns can be made between a typical drinking week and an intense drinking week. In a typical drinking week, similar to the findings of recent studies (Foster et al., 2015; Howard et al., 2015; Lau-Barraco et al., 2016), drinking was concentrated on Friday and Saturday. On each of those days, the participants ingested an average of five standard drinks. During an intense drinking week, drinking was spread out over weekdays and the weekend. A descriptive, yet striking, result was that the participants reported drinking an average of 7.55 standard drinks (106 g pure alcohol) on the heaviest Saturday outing, which increased to an average of 10 standard drinks (140 g pure alcohol) in men. Peaks of alcohol consumption during specific time-windows are associated with a higher likelihood of alcohol-related accidents (Foster et al., 2015), underscoring the need to center prevention efforts on reducing AU during these time-windows.

Our findings and other recent studies (Barry et al., 2016) suggested that alcohol was the entry-point substance for the majority of the participants. The onset of AU preceded the use of tobacco, which, in turn, preceded the use of marijuana (Gruber et al., 2012a). We identified substance-specific associations (Ohannessian et al., 2015). The early use of alcohol, tobacco, and marijuana was associated with a higher likelihood of consuming each of these substances. Despite this, an early drinking onset was significantly associated with a greater occurrence of all indicators of tobacco and marijuana use. Moreover, the effect sizes of the associations between early drinking onset and subsequent use of all three substances were larger than the effect of early tobacco or marihuana use on subsequent use of these substances. Altogether, these findings suggest a broader effect of alcohol initiation that heightens the risk of consuming alcohol and using other substances (Wagner et al., 2005; Hingson et al., 2008).

Compared with representative data from a national survey (SEDRONAR, 2010), we observed a decrease in the mean age of onset of the use of alcohol (∼15 vs. ∼17), tobacco (∼16 vs. ∼17), and marijuana (∼17 vs. ∼19). The findings from SEDRONAR were derived from a sample of 12–65 year old individuals, which may result in telescoping bias. Nonetheless, this notable decrease in the age of first use raises concerns about the significant effect of early substance use on future risk behaviors (Barry et al., 2016).

Similar to previous work that was mostly conducted in the United States (LaBrie et al., 2010a; Neighbors et al., 2011), we found a significant and positive association between the level of perceived approval and the use of alcohol, tobacco, and marijuana. Unsurprisingly, peer-related variables exerted a stronger effect than parent-related variables (Parsai et al., 2009). Interestingly, the role of parental norms was both substance- and sex-specific. Parents seemingly had a stronger impact on AU (only at the bivariate level) and marijuana use among men, whereas parents had a stronger impact on tobacco use among women. These findings suggest promising avenues for intervention and highlight the need to implement sex- and substance-specific programs. Injunctive norms, at least those for peer approval, can be altered by information-based manipulation (Prince and Carey, 2010; Ridout and Campbell, 2014). The findings also suggest that at least some risk factors for substance use may be universal. College life, social organization, and other important contextual factors (e.g., legal age to buy alcohol) are notably different between the United States and Argentina. Despite these differences, however, the present study indicated that certain vulnerability factors exert similar effects across these cultural contexts.

Students perceived their own drinking behaviors as lower than those of same-sex students. Women also perceived that opposite-sex students drank larger amounts of alcohol than they did. The latter more likely reflects sex differences in drinking behaviors, in which men reported greater AU than women. These findings support previous studies that suggested that students overestimated the drinking of their peers (Neighbors et al., 2008b).

Similar to previous work (Johnston et al., 2015), perceived risk was negatively associated with substance use, in which students who perceived alcohol, tobacco, and marijuana use as less risky reported greater use of each substance compared with students who perceived use as more risky. At the multivariate level, this cognitive variable significantly explained a greater frequency of binge drinking and tobacco and marijuana use. Notably, the effect size of perceived risk was greater for marijuana use at the bivariate and multivariate levels compared with alcohol and tobacco use. We also found a positive and significant association between perceived risk and injunctive norms. The students who perceived greater approval also perceived a lower risk associated with the use of that substance.

The present study has limitations. The cross-sectional design does not allow the determination of causal relationships between variables. A bidirectional rather than unilateral effect might underlie addictive behaviors, in which some conditions be a consequence rather than cause of drug exposure. This reciprocal association might be seen as an ongoing feedback cycle, in which lower risk perception promotes substance use, which, in turn, decreases the perceived risk of using that substance. Similar reciprocal relationships have been reported for impulsivity and drug use (Malmberg et al., 2013). Longitudinal studies that begin before direct contact with a substance are needed to further elucidate the role of risk and protective factors in the emergence of addictive behaviors. Another limitation of the present study was the assessment of descriptive norms only for alcohol and not for tobacco or marijuana.

Despite these limitations, a main contribution of this study was the description of substance use behaviors in a large sample of Argentinean college freshman (from many and different careers) and the relationship between these behaviors and the onset of substance use, descriptive and injunctive social norms, and perceived risk of using those substances. The findings suggest avenues of intervention in this target group. Programs that are directed toward delaying the onset of AU, which was shown to be a “gateway” drug with broader effects on the use of other substances, or modulating the perception of peers’ drug use and approval may be particularly useful among these individuals. Interventions that target the influence of perception of drug use may also be beneficial, particularly if the aim is to reduce marijuana use.

## Ethics Statement

This study was carried out in accordance with the recommendations of the National University of Cordoba’s internal review board with written informed consent from all subjects. All subjects gave written informed consent in accordance with the Declaration of Helsinki. The protocol was approved by the National Agency for Promotion of Science and Technology (FONCyT).

## Author Contributions

AP and RP designed the study, collected the data, and analyzed the data. JR helped designed the study and provided input in the data analysis. RP and AP wrote the initial version of the manuscript. All authors corrected and approved the final version of the MS.

## Conflict of Interest Statement

The authors declare that the research was conducted in the absence of any commercial or financial relationships that could be construed as a potential conflict of interest.
